# SARIFA as a new histopathological biomarker is associated with adverse clinicopathological characteristics, tumor-promoting fatty-acid metabolism, and might predict a metastatic pattern in pT3a prostate cancer

**DOI:** 10.1186/s12885-023-11771-9

**Published:** 2024-01-12

**Authors:** Johanna S. Enke, Matthias Groß, Bianca Grosser, Eva Sipos, Julie Steinestel, Phillip Löhr, Johanna Waidhauser, Constantin Lapa, Bruno Märkl, Nic G. Reitsam

**Affiliations:** 1 Nuclear Medicine, Faculty of Medicine, University of Augsburg, Augsburg, Germany; 2 Pathology, Faculty of Medicine, University of Augsburg, Augsburg, Germany; 3 Urology, Faculty of Medicine, University of Augsburg, Augsburg, Germany; 4 Hematology and Oncology, Faculty of Medicine, University of Augsburg, Augsburg, Germany

**Keywords:** Prostate cancer, Lipid metabolism, Biomarker study, Prognostic biomarker, pT3a prostate cancer

## Abstract

**Background:**

Recently, we introduced Stroma-AReactive-Invasion-Front-Areas (SARIFA) as a novel hematoxylin–eosin (H&E)-based histopathologic prognostic biomarker for various gastrointestinal cancers, closely related to lipid metabolism. To date, no studies on SARIFA, which is defined as direct tumor-adipocyte-interaction, beyond the alimentary tract exist. Hence, the objective of our current investigation was to study the significance of SARIFA in pT3a prostate cancer (PCa) and explore its association with lipid metabolism in PCa as lipid metabolism plays a key role in PCa development and progression.

**Methods:**

To this end, we evaluated SARIFA-status in 301 radical prostatectomy specimens and examined the relationship between SARIFA-status, clinicopathological characteristics, overall survival, and immunohistochemical expression of FABP4 and CD36 (proteins closely involved in fatty-acid metabolism). Additionally, we investigated the correlation between SARIFA and biochemical recurrence-free survival (BRFS) and PSMA-positive recurrences in PET/CT imaging in a patient subgroup. Moreover, a quantitative SARIFA cut-off was established to further understand the underlying tumor biology.

**Results:**

SARIFA positivity occurred in 59.1% (*n* = 178) of pT3a PCas. Our analysis demonstrated that SARIFA positivity is strongly associated with established high-risk features, such as R1 status, extraprostatic extension, and higher initial PSA values. Additionally, we observed an upregulation of immunohistochemical CD36 expression specifically at SARIFAs (*p* = 0.00014). Kaplan–Meier analyses revealed a trend toward poorer outcomes, particularly in terms of BRFS (*p* = 0.1). More extensive tumor-adipocyte interaction, assessed as quantity-dependent SARIFA-status on H&E slides, is also significantly associated with high-risk features, such as lymph node metastasis, and seems to be associated with worse survival outcomes (*p* = 0.16). Moreover, SARIFA positivity appeared to be linked to more distant lymph node and bone metastasis, although statistical significance was slightly not achieved (both *p* > 0.05).

**Conclusions:**

This is the first study to introduce SARIFA as easy-and-fast-to-assess H&E-based biomarker in locally advanced PCa. SARIFA as the histopathologic correlate of a distinct tumor biology, closely related to lipid metabolism, could pave the way to a more detailed patient stratification and to the development of novel drugs targeting lipid metabolism in pT3a PCa. On the basis of this biomarker discovery study, further research efforts on the prognostic and predictive role of SARIFA in PCa can be designed.

**Supplementary Information:**

The online version contains supplementary material available at 10.1186/s12885-023-11771-9.

## Background

Prostate cancer (PCa), which represents a significant portion of the global disease burden, is ranked third among all newly diagnosed malignancies worldwide and is the most common malignant tumor in men [1]. With demographic changes, the importance of PCa, primarily associated with increasing age, is expected to continue to grow. While many cases of PCa exhibit slow disease progression, a subset of patients develop aggressive forms of the disease with a higher risk of mortality. Therefore, the identification of prognostic biomarkers that can accurately predict disease outcomes is essential for guiding treatment strategies, facilitating appropriate interventions, and improving patient outcomes.

In recent years, there has been a shift in the focus of cancer research, particularly concerning PCa. The emphasis has been on understanding the intricate interplay between tumor cells and the surrounding stromal microenvironment, including adipocytes, both within the tumor itself and especially at the tumor invasion front [2–5].

Our research group recently introduced Stroma-AReactive-Invasion-Front-Areas (SARIFA) as a novel biomarker based on hematoxylin and eosin (H&E) staining. SARIFA is defined as direct contact between tumor cells and adipocytes at the tumor invasion front without the presence of intervening desmoplastic stroma and immune cells. This unique histological feature has shown promising prognostic relevance in colon and gastric cancer [6–9]. This biomarker can be assessed easily and rapidly without incurring additional costs, relying solely on routine histology [6–10].

There is evidence to suggest that SARIFA reflects the morphological manifestation of an aggressive tumor biology associated with alterations in lipid metabolism, such as the upregulation of FABP4 (fatty-acid binding protein 4) and CD36 (fatty-acid translocase, FAT) [7–9], which may not be limited to specific tumor types. Emerging evidence highlights the significant role of adipocytes and fatty acids in tumor progression [11, 12], particularly in the specific context of PCa [13, 14]. Periprostatic adipose tissue serves as an important energy resource for PCa cells [15] because these cells are characterized by a high uptake of fatty acids as metabolic substrates [16]. Notably, CD36, a major transporter for exogenous fatty acids into cells, is upregulated specifically at SARIFAs [7] and has already been established as a potential therapeutic target in PCa, as demonstrated by the efficacy of monoclonal antibodies against CD36 in patient-derived xenografts of pT3 PCa [17]. Should such treatment regimens be implemented in clinical practice, patient stratification based on robust biomarkers that reflect changes in lipid metabolism may become critical.

Building upon these observations, this study aims to investigate the potential of SARIFA-status as a biomarker in PCa.

## Methods

### Patients & ethics

We included 301 patients who underwent curative radical prostatectomy at the University Hospital Augsburg, Germany, between 2005 and 2020. The median age of the patients at the time of surgery was 68 years, with a range of 46–87 years. One individual had simultaneous but previously unknown metastatic disease at the time of initial diagnosis (SARIFA-positive case). We assembled the patient cohort through a retrospective database search of our institution’s internal laboratory information system. Detailed therapy data was not available for our cohort.

Follow-up data were obtained from Tumor Data Management at the Comprehensive Cancer Center Augsburg, University Hospital Augsburg. These data were supplemented with information from the patient files. The Bayerisches Landesamt für Gesundheit und Lebensmittelsicherheit, Bayerisches Krebsregister, Regionalzentrum Augsburg provided additional information on survival data.

The current study was conducted with the approval of the ethics committee of Ludwig Maximilians University of Munich, with reference project number 22–0960. The study adhered to the principles outlined in the Declaration of Helsinki.

### Histological workup and SARIFA assessment

Following each patient’s surgery, the entire prostatic resection specimen was promptly fixed in 4% buffered formalin for a minimum of 12 h. The specimens were then sectioned into complete transversal slices and embedded using a whole-mount sectioning approach for so-called large format histology to ensure orientation within large tissue cassettes [18]. This guarantees that the entire radical prostatovesiculectomy specimen of every patient including surrounding periprostatic soft tissue was completely processed and histologically investigated. Subsequently, 2 μm sections were cut from each specimen and stained with H&E (Merck, Darmstadt, Germany). We retrieved information regarding the primary pathological diagnosis, including grading and other relevant details, from reports signed by board-certified pathologists whenever available. Information about lymphatic (L), venous (V) and perineural invasion (Pn) was missing here in a small subset of cases. Extraprostatic extension (focal vs non-focal) was established according to Ball et al. [19], and also retrieved from pathologic reports of board-certified pathologists, whenever available. Risk groups for univariate Cox regression analysis were based on grade groups according to the ISUP 2014/WHO 2016 [20]. Hence, simplified risk groups are defined as follows: Grade Group 1 = 1; Grade Group 2 and 3 = 2; Grade Group 4 and 5 = 3.

**Fig. 1:**
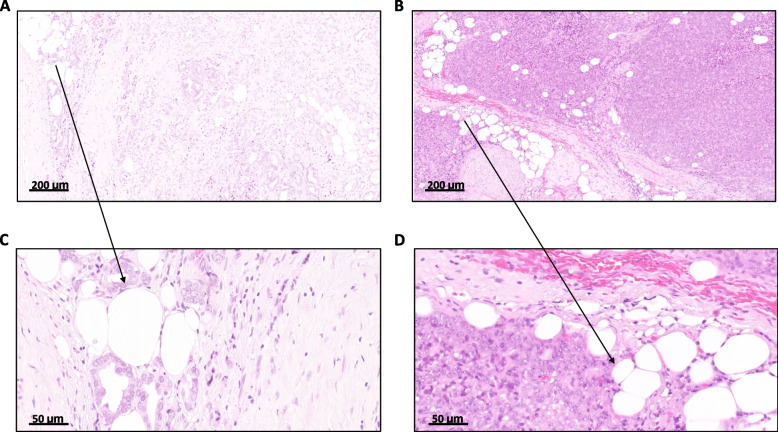
Definition of Stroma-AReactive-Invasion-Front-Areas (SARIFA) as haematoxylin & eosin (H&E) based histopathologic biomarker. SARIFA positivity is defined by the direct contact between adipocytes and tumor cells without intervening desmoplastic reaction, and SARIFA positivity is not restricted to a certain histology as it occurs in prostate cancer with glandular (**A**, **C**) as well as more solid (**B**, **D**) growth pattern. **A**, **B** Overviews of invasion front in periprostatic soft tissue, scale bar: 200 µm. **C**, **D** Close-ups of SARIFA-positive area with tumor cells directly adjacent to adipocytes, scale bar: 50 µm

The determination of SARIFA-status, which followed the methodology established in our previously published studies [6–10], was conducted by MG. In challenging or ambiguous cases, a double-head microscope was used to reevaluate the specimens together with BM, a board-certified pathologist, to establish a consensus diagnosis. SARIFA positivity was defined as the presence of an area within the tumor invasion front in the extraprostatic tissue in which one or more tumor glands and/or a group of at least five tumor cells were in direct contact with adipocytes, with no intervening inflammatory infiltrate or desmoplastic reaction (Fig. 1). In the general classification of SARIFA-status, if any area was classified as SARIFA-positive, the entire case was considered SARIFA-positive. This definition aligns with our previous biomarker definition [6–10] and offers the advantage of simplicity.

To investigate a potential quantitative aspect of the SARIFA phenomenon, we also introduced an alternative, quantitative SARIFA classification. In this latter approach, only cases that exhibited SARIFA positivity in at least one-third (≥ 33%) of all slides were classified as SARIFA-positive; otherwise, they were considered SARIFA-negative. Thus, the SARIFA-status of each large-format section slide was determined separately for each individual patient. The cut-off value for this quantitative classification was established using the R-package '*bhm'* to identify an unknown biomarker cut-off, as described by Chen et al. [21]. The estimated optimal cut-off threshold for SARIFA-status, based on the percentage of slides showing SARIFA positivity, was determined to be 0.318 (95% confidence interval (CI): 0.271–0.404). To simplify the approach, we defined SARIFA positivity as ≥ one-third of all slides (≥ 33%) showing SARIFA, which closely approximates 0.318 (31.8%) and yielded essentially identical results. This quantitative SARIFA classification was considered only for overall survival and clinicopathological characteristics, as the number of SARIFA-positive cases based on this cut-off was too small for other correlations, such as biochemical recurrence-free survival or PSMA-positive recurrences. Please refer to Figure S1 for further details regarding the biomarker threshold regression model used.

### Immunohistochemistry

All immunohistochemistry (IHC) was performed on the Leica Bond RX automated staining system (Leica, Wetzlar, Germany), following the automated standard IHC protocol optimized for use on this platform. For each SARIFA-positive and negative case, 2–4 μm thick, whole-slide, paraffin-embedded/FFPE sections were used. FFPE sections were deparaffinized in the instrument, followed by epitope retrieval for 20 min at 95 °C in EDTA (CD36) or 25 min at 100 °C in citrate (FABP4) and peroxidase block for 5 min at 25 °C.

Subsequently, the slides were incubated with antibodies against CD36 (fatty-acid translocase, Sigma HPA002018, rabbit polyclonal antibody, 1:50, Sigma, St. Louis, Missouri, USA) and against FABP4 (ab13979, fatty-acid-binding protein 4, rabbit polyclonal antibody, 1:200, Abcam, Cambridge, UK) diluted in Dako’s antibody diluting solution (Dako, DM830) for 32 min at 42 °C. Chromogen detection and hematoxylin counterstaining were performed using a bond polymer refine detection kit (Leica, Cat. No.: DS9800).

Similarly to Grosser et al. [7], regarding FABP4, the percentage of positive tumor cells was assessed.

For CD36 also the percentage of positive tumor cells was assessed. Both stainings were evaluated separately at the invasion front (IF) and tumor center (TC).

For immunohistochemistry only a representative subset of the cohort was used with comparably newer cases to ensure high tissue quality.

Representative slides of FABP4 and CD36 immunohistochemistry, as well as H&E stains, were digitized using a 3D Histech Panoramic Scan II (3D Histech, Budapest, Hungary).

### PSMA-PET imaging

We evaluated all included patients (n = 301) to determine whether they underwent a [^68^ Ga]Ga-PSMA-PET/CT scan as part of their clinical routine at the time of initial diagnosis or during follow-up when biochemical recurrence was present (PSMA-PET/CT performed: n = 57). We defined biochemical recurrence according to the criteria set by the American Urological Association, which requires a serum PSA level of ≥ 0.2 ng/mL, followed by a second confirmatory result [22]. [^68^ Ga]Ga-PSMA-PET/CT were performed on hybrid PET/CT scanner systems, either a Siemens Biograph mCT-S40 (Siemens Medical Solutions, Erlangen, Germany) or a Discovery MI (GE Medical Systems, Chicago, USA) system. Image acquisition and interpretation for PSMA-PET/CT were performed according to current guidelines [23].

During the routine clinical review, we examined all scans for the presence of PSMA-positive tumor lesions. Whenever applicable, we retrieved information about local recurrence, regional or distant lymph nodes, and hematogenic metastases (such as bone and lungs) from the nuclear medicine report.

### Statistical analysis

For statistical analysis, we employed IBM SPSS Statistics (Version 29.0.0.0, IBM, Armonk, NY, USA) and R, version 4.2.1 (R Foundation for Statistical Computing, Vienne, Austria). We used chi-square or Fisher’s exact tests to conduct hypothesis testing for differences between the relative frequencies of categorical variables and employed Mann–Whitney U tests to compare continuous variables. To compare the estimates of Kaplan–Meier survival probabilities, we utilized log-rank tests. The median follow-up duration was calculated using the reverse Kaplan–Meier method [24]. Hazard ratios (HRs) were estimated as relative risks through Cox proportional hazard models. We also performed multivariate Cox regression analysis including known relevant risk factors. HRs and 95% CIs were reported. We considered p-values < 0.05 as statistically significant.

## Results

### H&E-based SARIFA classification and its association with clinicopathological parameters

We determined SARIFA-status on all radical prostatectomy resection specimens in our cohort (*n* = 301). 59.1% (*n* = 178) of pT3a PCas were classified as SARIFA-positive, whereas 40.9% (*n* = 123) were classified as SARIFA-negative. Using the reverse Kaplan–Meier method [24], we estimated the median overall follow-up duration to be 69 months (95% CI, 55–83 months).

SARIFA positivity was significantly associated with microscopic residual tumor/positive surgical margin (R1 status, *p* = 0.039), non-focal extraprostatic extension (EPE, *p* < 0.001), and higher Gleason score as well as grade groups (each *p* = 0.015). Furthermore, SARIFA-positive PCas tended to have a higher frequency of lymphatic invasion (*p* = 0.079). Notably, when comparing the number of positive lymph nodes among all pN1 PCa cases, SARIFA-positive PCa cases exhibited a higher absolute count of positive lymph nodes (mean ± SD, SARIFA-positive: 2.12 ± 1.67; SARIFA-negative: 1.29 ± 0.66; *p* = 0.008), even though number of dissected lymph nodes did not differ between SARIFA-positive and SARIFA-negative cases (mean ± SD, SARIFA-positive: 16.81 ± 9.03; SARIFA-negative: 15.96 ± 9.50; *p* = 0.439). Preoperative initial prostate specific antigen (PSA) values were available for 211 patients (SARIFA-positive *n* = 123; SARIFA-negative *n* = 88). Here, patients with SARIFA-positive PCas showed higher initial PSA values (mean ± SD, SARIFA-positive: 17.41 ± 18.39 ng/ml, *p* = 0.004SARIFA-negative: 11.28 ± 7.80 ng/ml).

For a detailed overview of the clinicopathological characteristics of patients with SARIFA-positive and SARIFA-negative pT3a PCa, please refer to Table 1. For the association between SARIFA-status and grade groups according to ISUP 2014/WHO 2016 [20], we refer to Table S2.

**Table 1:** Clinicopathological characteristics of pT3a prostate carcinomas with regards to SARIFA-status

				SARIFA-status				
Variable		**All cases**		**SARIFA-positive**		**SARIFA-negative**		
		*n* = 301	100%	*n* = 178	59%	*n* = 123	41%	*p-value*
**Age in years, at surgery, median (range)**		68 (46–87)		68 (52–87)		68 (46–83)		0.336
**iPSA**^**a**^** (mean ± standard deviation), in ng/ml**		14.85 ± 15.19		17.41 ± 18.39		11.28 ± 7.80		**0.004**
**pN category**	pN0	212	70%	119	67%	93	76%	0.162
	pN1	87	29%	57	32%	30	24%	
	NA	2	1%	2	1%	0	0%	
**Gleason Score**	6	11	4%	3	2%	8	7%	**0.015**
	7	183	61%	107	60%	76	62%	
	8	48	16%	24	14%	24	20%	
	9	53	18%	39	22%	14	11%	
	10	6	2%	5	3%	1	1%	
**Lymphatic invasion**	L0	216	72%	128	72%	88	72%	0.079
	L1	26	9%	20	11%	6	5%	
	NA	59	20%	30	17%	29	24%	
**Vascular invasion**	V0	230	76%	140	79%	90	73%	0.137
	V1	12	4%	9	5%	3	2%	
	NA	59	20%	29	16%	30	24%	
**Perineural invasion**	Pn0	8	3%	4	2%	4	3%	0.449
	Pn1	252	84%	153	86%	99	81%	
	NA	41	14%	21	12%	20	16%	
**R status**	R0	172	57%	93	52%	79	64%	**0.039**
	R1	129	43%	85	48%	44	36%	
**Extraprostatic extension**	focal	158	52%	82	46%	76	62%	** < 0.001**
	non-focal	97	32%	39	22%	7	6%	
	NA	46	15%	57	32%	40	33%	

*p-*values that are statistically significant are highlighted in **bold**

^a^iPSA: initial prostate specific antigen values were only available for 211 patients (123 SARIFA-positive, 88 SARIFA-negative). *SARIFA* Stroma-AReactive-Invasion-Front-Areas, *pT* depth of invasion, *pN* lymph node status, *R status* residual tumor status; Extraprostatic extension according to Ball et al. [19]

We conducted a separate assessment of the SARIFA-status for each slide of each patient to gain a better understanding of the quantitative aspect of our biomarker SARIFA. Among SARIFA-positive PCa cases, an average of 25.4% of all slides showed SARIFA positivity (± SD, ± 16.9%, minimum: 4.6%; maximum: 100%). Furthermore, we employed a different, more stringent, quantity-dependent optimized cut-off for SARIFA positivity (≥ 1/3 of all slides). Upon applying this cut-off, only 15.6% (*n* = 47) of all cases were classified as SARIFA-positive. Interestingly, we observed a strong association between a more widespread SARIFA positivity and the presence of positive lymph nodes at the time of surgery (*p* = 0.004). SARIFA positivity was also associated with non-focal EPE (*p* = 0.005) and microscopic residual tumor/positive surgical margin (R1-status, *p* = 0.012). No significant association with grading was observed. Moreover, SARIFA positivity, according to our quantitative cut-off, was associated with a trend toward higher initial PSA values (quantitative SARIFA-positive n = 34; quantitative SARIFA-negative *n* = 177; mean ± SD, SARIFA-negative: 13.40 ± 11.52 ng/ml; SARIFA-positive: 22.42 ± 26.28 ng/ml, *p* = 0.091). For a detailed overview of clinicopathologic characteristics related to quantitative SARIFA-status, please refer to Table S1.

### Effect of SARIFA-status on outcome

**Fig. 2:**
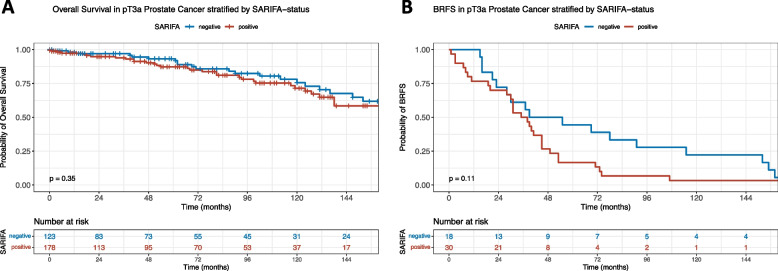
Kaplan–Meier curves regarding overall survival and BRFS of pT3a prostate cancer patients stratified by SARIFA-status**.** **A** SARIFA positivity is not associated with a statistically significant decreased overall survival in pT3a PCa patients (*p* = 0.35). **B** Regarding BRFS, SARIFA positivity seems to be associated with a trend towards high recurrence rates (*p* = 0.11) – however, patient number with BRFS was limited in our analysis. BRFS: biochemical recurrence-free survival, SARIFA: Stroma-AReactive-Invasion-Front-Areas pT: depth of invasion

Having previously demonstrated the prognostic relevance of SARIFA in gastric and colon cancer, we conducted a Kaplan–Meier analysis to evaluate the association between SARIFA-status and overall survival in our pT3a PCa cohort (Fig. 2A). Although the curves seemingly exhibited distinct separation, indicating a worse clinical course for SARIFA-positive cases, this association did not reach statistical significance (p = 0.35; HR of 1.290, 95% CI: 0.758–2.196). Similar trends were observed when using our more quantity-dependent definition of SARIFA positivity (≥ 1/3 of all slides SARIFA-positive; see Figure S2, *p* = 0.16; HR of 1.547, 95% CI: 0.832–2.876).

To further investigate the association between SARIFA-status and clinical outcomes, we focused on a subgroup of 48 patients who presented with biochemical recurrence in our clinic (no censored data). Notably, the Kaplan–Meier analysis again revealed a distinct separation of the survival curves between SARIFA-positive and SARIFA-negative (Fig. 2B) without reaching statistical significance (*p* = 0.11; HR of 1.632, 95% CI: 0.883–3.015), suggesting a potential impact of SARIFA-status on biochemical recurrence-free survival.

We conducted a univariate Cox regression analysis to assess the prognostic significance of various pathologic risk factors and our novel biomarker SARIFA in pT3a PCa. Among the examined factors, including initial PSA, age (> 65), extraprostatic extension (focal vs non-focal), pN status (pN0 vs pN1), R status (R0 vs R1), lymphatic invasion (no vs yes), vascular invasion (no vs yes), perineural invasion (no vs yes), and risk group, only higher initial PSA values were associated with a slightly decreased overall survival (*p* < 0.001, HR of 1.031, 95% CI: 1.014–1.048). The results of the univariate Cox regression analysis are summarized in Table 2. This statistically significant association between initial PSA values and overall survival remained true upon multivariate analysis (see Table S3; as only one of the included variables did show statistical significance upon univariate Cox regression, results of our multivariate model should be interpreted carefully due to potential overfitting of the model).

**Table 2:** Univariate Cox regression analysis regarding overall survival in pT3a prostate cancer

	**Univariate Cox Regression**	
**Overall Survival**	Hazard Ratio (95% CI)	*p-*value
iPSA	1.031 (1.014–1.048)	** < 0.001**
Age > 65	1.743 (0.879–3.454)	0.112
Extraprostatic extension (focal vs non-focal)	0.718 (0.293–1.758)	0.469
pN (pN0 vs pN1)	0.768 (0.413–1.430)	0.405
R status (R0 vs R1)	1.003 (0.591–1.704)	0.99
Lymphatic invasion (no vs yes)	1.097 (0.459–2.621)	0.835
Vascular invasion (no vs yes)	1.631 (0.386–6.883)	0.506
Perineural invasion	2.772 (0.380–20.245)	0.315
*Risk group	1.227 (0.765–1.969)	0.395
SARIFA (negative vs positive)	1.290 (0.758–2.196)	0.348

*p*-values that are statistically significant are highlighted in **bold**

*iPSA: initial prostate specific antigen values were only available for 211 patients. *SARIFA* Stroma-AReactive-Invasion-Front-Areas, *pT* depth of invasion, *pN* lymph node status, *R status* residual tumor status; Extraprostatic extension according to Ball et al. [19]; *Simplified risk groups are defined as: Grade Group 1 = 1; Grade Group 2 and 3 = 2; Grade Group 4 and 5 = 3 [20]

### Expression of fatty-acid metabolism-associated proteins at SARIFAs

**Fig. 3:**
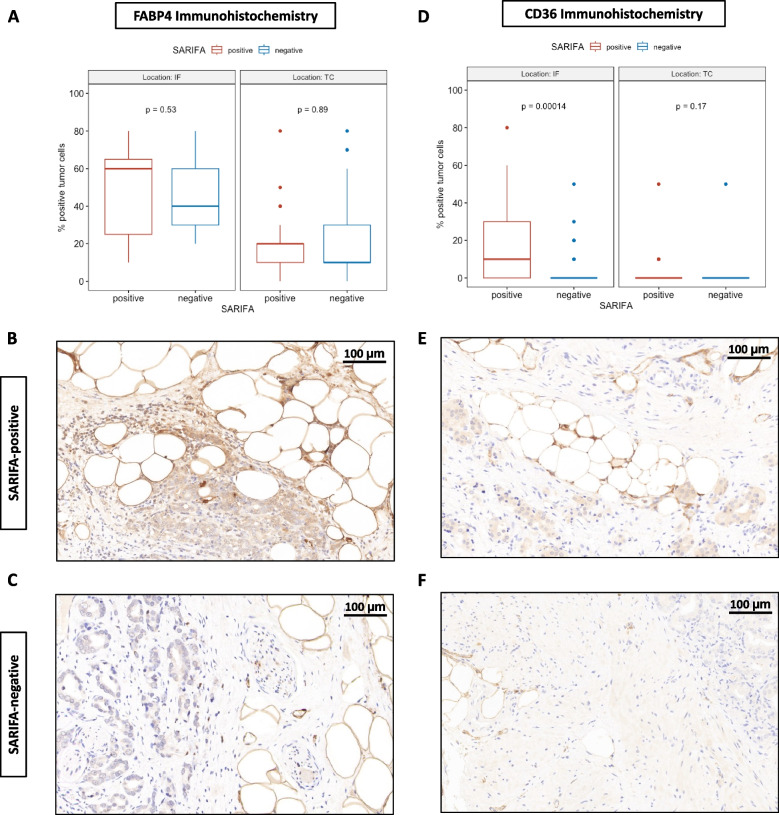
Immunohistochemical analysis of FABP4 and CD36 expression with regards to SARIFA-status. **A** No SARIFA-dependent differences regarding FABP4 expression could be observed (IF: *p* = 0.53, TC: *p* = 0.89). **D** CD36 immunohistochemistry revealed CD36 upregulation, specifically at the invasion front (IF: *p* = 0.0012, TC: *p* = 0.4). **B**, **E** Exemplary images of SARIFA-positive cases. **C**, **F** Exemplary images of SARIFA-negative cases, scale bars: 100 µm. SARIFA: Stroma-AReactive-Invasion-Front-Areas FABP4: fatty-acid binding protein 4, CD36: cluster of differentiation 36, fatty-acid translocase, IF: invasion front, TC: tumor center

In a previous study, we successfully demonstrated upregulation of FABP4 and CD36 expression, two proteins known to be involved in lipid metabolism, specifically at SARIFAs [7]. To further investigate this phenomenon, we conducted immunohistochemical staining for these two proteins in a subset of our cohort (FABP4: *n* = 57; CD36: *n* = 59). While no SARIFA-dependent changes were observed in FABP4 protein expression (IF: *p* = 0.53; TC: *p* = 0.89), SARIFA-positive cases exhibited a statistically significant increase in CD36 expression at the invasion front (*p* = 0.00014). No differences regarding CD36 expression could be observed in the tumor center (*p* = 0.17). The results of the immunohistochemical staining, as well as representative images, are presented in Fig. 3. As most research on FABP4 and CD36 pertains to obesity, we compared the body mass index (BMI) of 30 SARIFA-positive and 30 SARIFA-negative patients. However, no SARIFA-dependent differences in BMI were detected (mean ± SD, SARIFA-positive: 28.73 ± 3.85; SARIFA-negative: 27.99 ± 4.30, *p* = 0.487).

### Exploratory correlation of SARIFA-status and PSMA-PET Results

Out of our whole cohort, 57 patients underwent follow-up [^68^ Ga]Ga-PSMA-PET/CT imaging in nuclear medicine (59.6% SARIFA-positive; 40.4% SARIFA-negative). To further understand the relevance of SARIFA-status to recurrences, we correlated PSMA-PET/CT findings with SARIFA-status. There was no significant difference in local recurrence rates between the SARIFA-positive and SARIFA-negative groups (*p* = 0.877). However, SARIFA positivity seemed to be associated with higher rates of regional and distant lymph node metastasis as well as higher frequency of bone metastasis. The difference in regional lymph node involvement between the SARIFA-positive and SARIFA-negative groups approached but did not reach statistical significance (*p* = 0.140). The difference in distant lymph node metastasis between SARIFA-positive and SARIFA-negative groups showed a clear trend and just failed to show statistical significance (*p* = 0.066). The difference in bone metastasis between SARIFA-positive and SARIFA-negative groups also approached statistical significance (*p* = 0.061). No significant differences were observed in pulmonary metastasis or soft tissue involvement. These findings are summarized in Table 3.

**Table 3:** Relationship between Stroma-AReactive-Invasion-Front-Areas and PSMA-positive recurrences in PET/CT imaging

		**SARIFA-positive**		**SARIFA-negative**		
*n* in total: 57		*n*	in %	*n*	in %	
*PSMA-positive Recurrence*		34	59.6	23	40.4	*p-*value
Local	no	20	58.8	14	60.9	0.877
	yes	14	41.2	9	39.1	
Regional Lymph Nodes	no	22	64.7	19	82.6	0.140
	yes	12	35.3	4	17.4	
Distant Lymph Nodes	no	29	85.3	23	100.0	0.066
	yes	5	14.7	0	0.0	
Bone	no	22	64.7	20	87.0	0.061
	yes	12	35.3	3	13.0	
Pulmonary	no	32	94.1	22	95.7	0.799
	yes	2	5.9	1	4.3	
Soft Tissue	no	34	100.0	23	100.0	/
	yes	0	0.0	0	0.0	

*p*-values that are statistically significant are highlighted in **bold**

Distant lymph nodes were defined as all extra-pelvic lymph node manifestations

*PET/CT* Positron emission tomography/Computed tomography, *PSMA* prostate-specific membrane antigen, *SARIFA* Stroma-AReactive-Invasion-Front-Areas

**Fig. 4:**
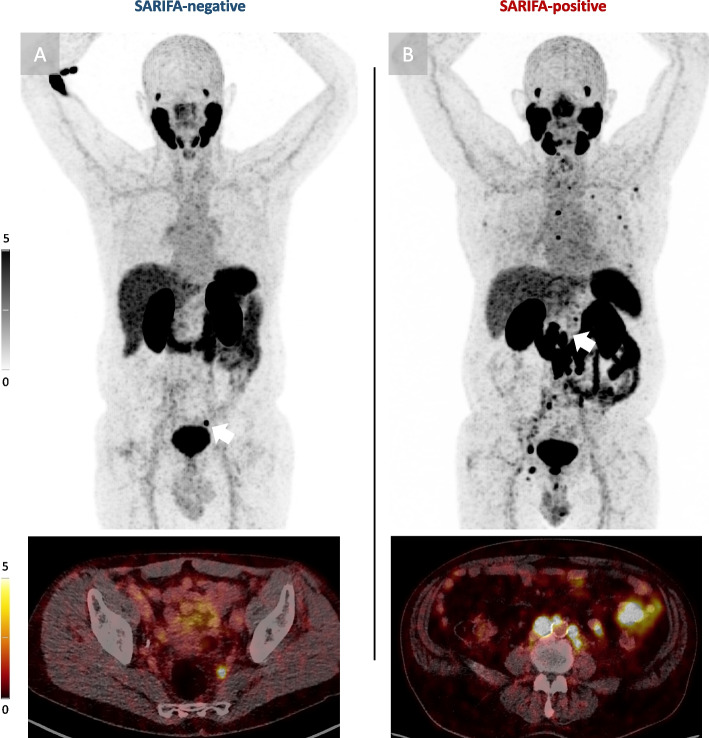
Exemplary maximum intensity projections of PSMA-PET/CT at point of first biochemical recurrence. **A** SARIFA-negative pT3a prostate cancer patient with a PSMA-positive iliaco-internal lymph node (marked by white arrow). **B** SARIFA-positive pT3a prostate cancer patient with PSMA-positive regional as well as distant lymph node (marked by white arrow) involvement as well as several bone metastasis. Distant lymph nodes were defined as all extra-pelvic lymph node manifestations. pT: depth of invasion, PET/CT: Positron emission tomography/Computed tomography, PSMA: prostate-specific membrane antigen, SARIFA: Stroma-AReactive-Invasion-Front-Areas SUV: standardized uptake volume

PSMA-PET/CT imaging is paradigmatically visualized for a SARIFA-positive and SARIFA-negative patient with PSMA-positive recurrences in Fig. 4.

## Discussion

Even though PCa is generally associated with good survival rates, higher T stages, such as pT3a with extension to the periprostatic soft tissue, are characterized by a more aggressive course of disease with higher recurrence rates and poorer outcomes [25]. Hence, it is necessary to develop new biomarkers that focus specifically on those cases with aggressive tumor biology.

To address this clinical need, we applied our newly established H&E-based biomarker SARIFA to a cohort of 301 pT3a PCas. In this study, we investigated the hypothesis that SARIFA positivity, defined as the presence of direct contact between tumor cells and adipocytes, is in PCa, just as in gastric and colon cancer, associated with high-risk features, a poor prognosis, and upregulation of proteins associated with lipid metabolism [6–9].

Interestingly, SARIFA positivity was statistically associated with positive surgical margins (R1 status), non-focal EPE, and higher initial PSA values, all known high-risk features [19, 26–28]. However, compared to status of surgical margins [27] and EPE [29], SARIFA evaluation offers the advantage of extremely high interobserver agreement [6, 7]. Because occasionally the SARIFA phenomenon is pronounced only focally, while other times it is widespread, similar to EPE [26], we applied a more quantitative approach by using an optimized cut-off of more than one-third of slides with SARIFAs for a quantity-dependent SARIFA-status classification. Here, indeed, widespread SARIFA positivity was strongly associated with positive lymph nodes, non-focal EPE, and positive surgical margins. However, no significant association with grading was observed. Nevertheless, we believe that our binary SARIFA classification, which is highly prognostic in colon and gastric cancer, offers a great advantage because its assessment is clear, easy, and fast, making it suitable for clinical implementation. Altogether, these findings suggest that SARIFA positivity may serve as a potential indicator of aggressive PCa characteristics, similar to our findings with other entities [6–10]. Moreover, it can be assumed that the SARIFA phenomenon is not restricted to tumors of the alimentary tract, but rather reflects a general tumor biological principle.

The association between SARIFA-status and overall survival in pT3a PCa was assessed using Kaplan–Meier analyses. Although the log-rank p-values were not statistically significant (*p* = 0.35 and *p* = 0.16, respectively), there was a noticeable separation of survival curves, indicating at least a potential trend toward slightly worse survival in SARIFA-positive cases. Further investigation in a subgroup of 48 patients with biochemical recurrence showed a stronger difference, with a trend-wise higher rate of recurrences in SARIFA-positive cases without; however, again not reaching statistical significance (*p* = 0.1). In this context, it must be underlined that in univariate Cox regression analysis, except for initial PSA values, none of the broadly accepted pathologic risk factors demonstrated significant associations with overall survival in our cohort. This may explain the lack of significance of SARIFA. Therefore, we assume that our results at least suggest a potential impact of SARIFA on biochemical recurrence-free survival, which is the more common endpoint in PCa biomarker studies [19, 26, 27, 30], but was unfortunately only partly available for our cohort. Of course, this findings have to interpreted with caution.

Just recently, different deep-learning (DL) models trained to predict survival/lymph node metastasis from H&E slides in gastric and colorectal cancer have also appreciated the important role of tumor-adipocyte colocalization as so-far underappreciated morphological feature on prognosis of cancer patients [31–35]. Even though many publications highlight the important role of DL in identifying prostate cancer on biopsy material [36, 37], not many DL models have been deployed on resection specimens [38] with carcinoma cells invading into the extraprostatic tissue. Therefore, it is conceivable that future studies deploying DL on prostate cancer resection specimens for survival prediction allow further insights into tumor-adipocyte interaction as relevant morphologic feature – in analogy to what we have seen in colorectal cancer [31–34]. In this context, it should be noted that DL-based radiogenomic approaches will presumably also contribute to a better understanding of the tumor-stroma interaction in PCa in the near future [39, 40].

Given the ample evidence for the major role of lipid metabolism in tumor progression in cancer in general, but also in the context of PCa [11, 12], we studied the immunohistochemical protein expression of FABP4 and CD36, both key players in fatty-acid metabolism. In previous studies, we demonstrated upregulation of FABP4 and CD36 in SARFIFA-positive gastric and colorectal cancers by investigating spatial and bulk RNA-data as well as immunohistochemical protein expression [7–9]. Moreover, both FABP4 [41, 42] and CD36 [17] have already been linked to PCa progression. In our current study, we also show an upregulation of CD36 at SARIFAs in pT3a PCa, which is promising, as the efficacy of monoclonal antibodies against CD36 in the specific context of pT3a PCas has already been demonstrated by deploying patient-derived xenografts [17]. If such treatments find their way into the clinic, SARIFA-status could serve as a tailored biomarker, as SARIFA is likely to reflect a certain lipid-driven tumor biology.

These promising findings highlight the need for more studies investigating the role of tumor-adipocyte interaction in PCas by novel techniques, such as spatial transcriptomics [43] or single-cell RNA-sequencing (scRNA-seq), preferably in the specific context of SARIFA. Just recently, studies based on scRNA-seq could prove that the upregulation of fatty-acid metabolism seems not only to be a key feature along PCa progression [44] but also may have therapeutic implications with regards to androgen deprivation therapy (ADT) [45].

Even though most research on lipid metabolism and PCa progression has been considered with obesity as the underlying phenomenon [46], we could not find any SARIFA-dependent changes in BMI in our current study, similar to results for gastric cancer [7]. This finding, again, indicates that the SARIFA phenomenon is not driven or supported by obesity. Still it should be kept in mind that obesity seems to be a relevant clinical risk factor in PCa, yet again supporting the important role of adipose tissue and lipid metabolism in PCa progression [47, 48].

Additionally, we investigated whether SARIFA positivity is associated with a different PSMA-PET positive recurrence pattern. Here, we found a clear trend towards more distant lymph nodes and bone metastases, again slightly failing statistical significance. In other contexts such as for histological subtypes or molecular alterations it has been already shown that certain characteristics of the primary tumor can determine a metastatic pattern [49–51]. Therefore, based on our first promising findings in this comparably small exploratory approach, we believe linking SARIFA as histopathologic biomarker to a distinct metastatic spread should be studied further in upcoming research efforts.

As it is also known that (neo-)adjuvant ADT may alter the tumor microenvironment in prostate cancer patients [52], further studies should also include therapy data to assess the effect and role of ADT on tumor-adipocyte interaction and SARIFA.

Despite observing distinct survival curves and potential trends in SARIFA-positive PCa, the lack of statistical significance hints at the main limitations of this study. First, the sample size of the cohort, especially in the subgroup with biochemical recurrence and PSMA-PET/CT imaging, was relatively small, which may have compromised the ability to detect significant differences. Additionally, the follow-up period might not have been sufficiently long to capture the full impact of SARIFA-status on overall survival. This warrants caution in interpreting the results. Moreover, BRFS seems to be the more suitable endpoint for establishing biomarkers in PCa.

## Conclusion

In conclusion, this is the first study to evaluate SARIFA as a new cancer biomarker beyond the alimentary tract. Significant associations with established biomarkers indicate a prognostic potential for SARIFA. Moreover, trends toward a higher and different metastatic potential of SARIFA-positive PCa further suggest a tumor-supporting effect of the cancer–adipocyte interplay. Our results may warrant a much larger, preferably multicentric, study with long follow-up and BRFS as an additional or primary endpoint.

### Supplementary Information

**Additional file 1: Figure S1.** Biomarker threshold regression model for identifying optimal cut-off of SARIFA-positive slides (percentage) for our quantitative approach. **Figure S2.** Kaplan-Meier curves regarding overall survival of pT3a prostate cancer patients stratified by quantitative and optimized SARIFA-status. **Table S1.** Clinicopathological characteristics of pT3a prostate carcinomas with regards to quantitative (≥1/3 of all slides) SARIFA-status. **Table S2.** Association between grade groups and SARIFA-status in pT3a grostate carcinomas. **Table S3.** Multivariate Cox regression analysis regarding overall survival in pT3a prostate cancer.

## Data Availability

Further information on the datasets can be obtained from the corresponding author (NGR) on reasonable request and in restricted form due to privacy reasons. However, all relevant data generated during this study is included in this article.

## Abbreviations

PCa: Prostate cancer
SARIFA: Stroma-AReactive-Invasion-Front-Areas
H&E: Hematoxylin and eosin
FABP4: Fatty-acid binding protein 4
CD36: Fatty-acid translocase
IHC: Immunohistochemistry
FFPE: Formalin-fixed paraffin-embedded
IF: Invasion front
TC: Tumor center
HR: Hazard ratio
CI: Confidence interval
EPE: Extraprostatic extension
SD: Standard deviation
PSA: Prostate specific antigen
BMI: Body mass index
DL: Deep-learning
ADT: Androgen deprivation treatment

## References

[1] Sung H, Ferlay J, Siegel RL, Laversanne M, Soerjomataram I, Jemal A (2021). Global cancer statistics 2020: GLOBOCAN estimates of incidence and mortality worldwide for 36 cancers in 185 countries. CA Cancer J Clin.

[2] Shiao SL, Chu GC, Chung LWK (2016). Regulation of prostate cancer progression by the tumor microenvironment. Cancer Lett.

[3] Bahmad HF, Jalloul M, Azar J, Moubarak MM, Samad TA, Mukherji D (2021). Tumor microenvironment in prostate cancer: Toward identification of novel molecular biomarkers for diagnosis, prognosis, and therapy development. Front Genet.

[4] Cancel M, Pouillot W, Maheo K, Fontaine A, Crottes D, Fromont G (2022). Interplay between prostate cancer and adipose microenvironment: A complex and flexible scenario. Int J Mol Sci.

[5] Lasorsa F, di Meo NA, Rutigliano M, Ferro M, Terracciano D, Tataru OS (2023). Emerging hallmarks of metabolic reprogramming in prostate cancer. Int J Mol Sci.

[6] Martin B, Grosser B, Kempkens L, Miller S, Bauer S, Dhillon C (2021). Stroma AReactive invasion front areas (SARIFA)-A new easily to determine biomarker in colon cancer-results of a retrospective study. Cancers (Basel).

[7] Grosser B, Gluckstein M, Dhillon C, Schiele S, Dintner S, VanSchoiack A (2022). Stroma AReactive invasion front areas (SARIFA) - a new prognostic biomarker in gastric cancer related to tumor-promoting adipocytes. J Pathol.

[8] Reitsam NG, Grozdanov V, Löffler CML, et al. Novel biomarker SARIFA in colorectal cancer: highly prognostic, not genetically driven and histologic indicator of a distinct tumor biology. Cancer Gene Ther. 2023. 10.1038/s41417-023-00695-y, https://pubmed.ncbi.nlm.nih.gov/37990064/. Online ahead of print. 10.1038/s41417-023-00695-y PMC10874891 37990064

[9] Grosser B, Heyer CM, Austgen J, et al. Stroma AReactive Invasion Front Areas (SARIFA) proves prognostic relevance in gastric carcinoma and is based on a tumor-adipocyte interaction indicating an altered immune response. Gastric Cancer. 2023. 10.1007/s10120-023-01436-8, https://pubmed.ncbi.nlm.nih.gov/37874427/. Online ahead of print. 10.1007/s10120-023-01436-8 PMC10761465 37874427

[10] Reitsam NG, Markl B, Dintner S, Sipos E, Grochowski P, Grosser B (2023). Alterations in natural killer cells in colorectal cancer patients with stroma AReactive invasion front areas (SARIFA). Cancers (Basel).

[11] Mukherjee A, Bilecz AJ, Lengyel E (2022). The adipocyte microenvironment and cancer. Cancer Metastasis Rev.

[12] Vasseur S, Guillaumond F (2022). Lipids in cancer: A global view of the contribution of lipid pathways to metastatic formation and treatment resistance. Oncogenesis.

[13] Laurent V, Guerard A, Mazerolles C, Le Gonidec S, Toulet A, Nieto L (2016). Periprostatic adipocytes act as a driving force for prostate cancer progression in obesity. Nat Commun.

[14] Su F, Daquinag AC, Ahn S, Saha A, Dai Y, Zhao Z (2021). Progression of prostate carcinoma is promoted by adipose stromal cell-secreted CXCL12 signaling in prostate epithelium. NPJ Precis Oncol.

[15] Balaban S, Nassar ZD, Zhang AY, Hosseini-Beheshti E, Centenera MM, Schreuder M (2019). Extracellular fatty acids are the major contributor to lipid synthesis in prostate cancer. Mol Cancer Res.

[16] Liu Y, Zuckier LS, Ghesani NV (2010). Dominant uptake of fatty acid over glucose by prostate cells: A potential new diagnostic and therapeutic approach. Anticancer Res.

[17] Watt MJ, Clark AK, Selth LA, Haynes VR, Lister N, Rebello R, et al. Suppressing fatty acid uptake has therapeutic effects in preclinical models of prostate cancer. Sci Transl Med. 2019;11(478):eaau5758. 10.1126/scitranslmed.aau5758. 10.1126/scitranslmed.aau5758 30728288

[18] Cimadamore A, Cheng L, Lopez-Beltran A, Mazzucchelli R, Luciano R, Scarpelli M (2021). Added clinical value of whole-mount histopathology of radical prostatectomy specimens: a collaborative review. Eur Urol Oncol.

[19] Ball MW, Partin AW, Epstein JI (2015). Extent of extraprostatic extension independently influences biochemical recurrence-free survival: Evidence for further pT3 subclassification. Urology.

[20] Humphrey PA, Moch H, Cubilla AL, Ulbright TM, Reuter VE (2016). The 2016 WHO classification of tumours of the urinary system and male genital organs-part B: prostate and bladder tumours. Eur Urol.

[21] Chen BE, Jiang W, Tu D. A hierarchical bayes model for biomarker subset effects in clinical trials. Comput Stat Data Anal. 2014;71:324–34. Available from: https://www.sciencedirect.com/science/article/pii/S0167947313002004.

[22] Eastham JA, Auffenberg GB, Barocas DA, Chou R, Crispino T, Davis JW (2022). Clinically localized prostate cancer: AUA/ASTRO guideline, part II: principles of active surveillance, principles of surgery, and follow-up. J Urol.

[23] Fendler WP, Eiber M, Beheshti M, Bomanji J, Calais J, Ceci F, et al. PSMA PET/CT: Joint EANM procedure guideline/SNMMI procedure standard for prostate cancer imaging 2.0. Eur J Nucl Med Mol Imaging. 2023;50(5):1466–86. 10.1007/s00259-022-06089-w PMC10027805 36604326

[24] Schemper M, Smith TL (1996). A note on quantifying follow-up in studies of failure time. Control Clin Trials.

[25] Epstein JI, Carmichael MJ, Pizov G, Walsh PC (1993). Influence of capsular penetration on progression following radical prostatectomy: a study of 196 cases with long-term followup. J Urol.

[26] Park CK, Chung YS, Choi YD, Ham WS, Jang WS, Cho NH (2021). Revisiting extraprostatic extension based on invasion depth and number for new algorithm for substaging of pT3a prostate cancer. Sci Rep.

[27] van der Kwast TH, Collette L, Van Poppel H, Van Cangh P, Vekemans K, DaPozzo L (2006). Impact of pathology review of stage and margin status of radical prostatectomy specimens (EORTC trial 22911). Virchows Arch.

[28] Han M, Partin AW, Zahurak M, Piantadosi S, Epstein JI, Walsh PC (2003). Biochemical (prostate specific antigen) recurrence probability following radical prostatectomy for clinically localized prostate cancer. J Urol.

[29] Evans AJ, Henry PC, Van der Kwast TH, Tkachuk DC, Watson K, Lockwood GA (2008). Interobserver variability between expert urologic pathologists for extraprostatic extension and surgical margin status in radical prostatectomy specimens. Am J Surg Pathol.

[30] Sung M, Lin H, Koch MO, Davidson DD, Cheng L (2007). Radial distance of extraprostatic extension measured by ocular micrometer is an independent predictor of prostate-specific antigen recurrence: A new proposal for the substaging of pT3a prostate cancer. Am J Surg Pathol.

[31] Foersch S, Glasner C, Woerl A, Eckstein M, Wagner D, Schulz S, et al. Multistain deep learning for prediction of prognosis and therapy response in colorectal cancer. Nat Med. 2023;29:430–9. 10.1038/s41591-022-02134-1 36624314

[32] Wulczyn E, Steiner DF, Moran M, Plass M, Reihs R, Tan F (2021). Interpretable survival prediction for colorectal cancer using deep learning. NPJ Digit Med.

[33] L’Imperio V, Wulczyn E, Plass M, Muller H, Tamini N, Gianotti L, et al. Pathologist validation of a machine learning-derived feature for colon cancer risk stratification. JAMA Netw Open. 2023;6(3): e2254891. 10.1001/jamanetworkopen.2022.54891 PMC10015309 36917112

[34] Krogue JD, Azizi S, Tan F, Flament-Auvigne I, Brown T, Plass M (2023). Predicting lymph node metastasis from primary tumor histology and clinicopathologic factors in colorectal cancer using deep learning. Commun Med (Lond).

[35] Muti HS, Rocken C, Behrens H, Loffler CML, Reitsam NG, Grosser B, et al. Deep learning trained on lymph node status predicts outcome from gastric cancer histopathology: a retrospective multicentric study. Eur J Cancer. 2023;194:113335. 10.1016/j.ejca.2023.113335 37862795

[36] Perincheri S, Levi AW, Celli R, Gershkovich P, Rimm D, Morrow JS, et al. An independent assessment of an artificial intelligence system for prostate cancer detection shows strong diagnostic accuracy. Mod Pathol. 2021;34(8):1588–95. 10.1038/s41379-021-00794-x PMC8295034 33782551

[37] Sandeman K, Blom S, Koponen V, Manninen A, Juhila J, Rannikko A, et al. AI model for prostate biopsies predicts cancer survival. Diagnostics (Basel). 2022;12(5):1031. 10.3390/diagnostics12051031. 10.3390/diagnostics12051031 PMC9139241 35626187

[38] Melo PAdS, Estivallet CLN, Srougi M, Nahas WC, Leite KRM. Detecting and grading prostate cancer in radical prostatectomy specimens through deep learning techniques. Clinics (Sao Paulo). 2021;76:e3198. 10.6061/clinics/2021/e3198 PMC8527555 34730614

[39] Ferro M, de Cobelli O, Vartolomei MD, Lucarelli G, Crocetto F, Barone B (2021). Prostate cancer radiogenomics-from imaging to molecular characterization. Int J Mol Sci.

[40] Ferro M, de Cobelli O, Musi G, Del Giudice F, Carrieri G, Busetto GM (2022). Radiomics in prostate cancer: an up-to-date review. Ther Adv Urol.

[41] Uehara H, Takahashi T, Oha M, Ogawa H, Izumi K (2014). Exogenous fatty acid binding protein 4 promotes human prostate cancer cell progression. Int J Cancer.

[42] Huang M, Narita S, Inoue T, Koizumi A, Saito M, Tsuruta H (2017). Fatty acid binding protein 4 enhances prostate cancer progression by upregulating matrix metalloproteinases and stromal cell cytokine production. Oncotarget.

[43] Beechem JM (2020). High-plex spatially resolved RNA and protein detection using digital spatial profiling: a technology designed for immuno-oncology biomarker discovery and translational research. Methods Mol Biol.

[44] Zhang B, Wang S, Fu Z, Gao Q, Yang L, Lei Z (2023). Single-cell RNA sequencing reveals intratumoral heterogeneity and potential mechanisms of malignant progression in prostate cancer with perineural invasion. Front Genet.

[45] Horning AM, Wang Y, Lin C, Louie AD, Jadhav RR, Hung C (2018). Single-cell RNA-seq reveals a subpopulation of prostate cancer cells with enhanced cell-cycle-related transcription and attenuated androgen response. Cancer Res.

[46] Scaglia N, Frontini-Lopez YR, Zadra G. Prostate cancer progression: as a matter of fats. Front Oncol. 2021;11:719865. 10.3389/fonc.2021.719865 PMC8353450 34386430

[47] de Cobelli O, Terracciano D, Tagliabue E, Raimondi S, Galasso G, Cioffi A (2015). Body mass index was associated with upstaging and upgrading in patients with low-risk prostate cancer who met the inclusion criteria for active surveillance. Urol Oncol.

[48] Ferro M, Terracciano D, Musi G, de Cobelli O, Vartolomei MD, Damiano R, et al. Increased body mass index is a risk factor for poor clinical outcomes after radical prostatectomy in men with international society of urological pathology grade group 1 prostate cancer diagnosed with systematic biopsies. Urol Int. 2022;106(1):75–82. 10.1159/000516680 34167120

[49] Hugen N, van de Velde CJH, de Wilt JHW, Nagtegaal ID (2014). Metastatic pattern in colorectal cancer is strongly influenced by histological subtype. Ann Oncol.

[50] Wu SG, Zhang WW, Sun JY, Li FY, Lin Q, He ZY. Patterns of distant metastasis between histological types in esophageal cancer. Front Oncol. 2018;08(8):302. 10.3389/fonc.2018.00302 PMC6092597 30135855

[51] Nguyen B, Fong C, Luthra A, Smith SA, DiNatale RG, Nandakumar S, et al. Genomic characterization of metastatic patterns from prospective clinical sequencing of 25,000 patients. Cell. 2022;185(3):563,575.e11. 10.1016/j.cell.2022.01.003 PMC9147702 35120664

[52] Mao Y, Xu Y, Chang J, Chang W, Lv Y, Zheng P (2022). The immune phenotypes and different immune escape mechanisms in colorectal cancer. Front Immunol.

