# Supplementary material for: SARIFA as a new histopathological biomarker is associated with adverse clinicopathological characteristics, tumor-promoting fatty-acid metabolism, and might predict a metastatic pattern in pT3a prostate cancer
Source: BMC Cancer. 2024 Jan 12;24:65. doi: 10.1186/s12885-023-11771-9 (PMC10785487; doi:10.1186/s12885-023-11771-9)
Supplement: Supplementary file 1 — Additional file 1: Figure S1. Biomarker threshold regression model for identifying optimal cut-off of SARIFA-positive slides (percentage) for our quantitative approach. Figure S2. Kaplan-Meier curves regarding overall survival of pT3a prostate cancer patients stratified by quantitative and optimized SARIFA-status. Table S1. Clinicopathological characteristics of pT3a prostate carcinomas with regards to quantitative (≥1/3 of all slides) SARIFA-status. Table S2. Association between grade groups and SARIFA-status in pT3a grostate carcinomas. Table S3. Multivariate Cox regression analysis regarding overall survival in pT3a prostate cancer. [file 12885_2023_11771_MOESM1_ESM.docx]

Supplementary Information for

**SARIFA as a new histopathological biomarker is associated with adverse clinicopathological characteristics, tumor-promoting fatty-acid metabolism, and might predict a metastatic pattern in pT3a prostate cancer**

Johanna S. Enke,^1^ Matthias Groß,^2^ Bianca Grosser,^2^ Eva Sipos,^2^ Julie Steinestel,^3^ Phillip Löhr,^4^ Johanna Waidhauser,^4^ Constantin Lapa,^1^ Bruno Märkl,^2^ *Nic G. Reitsam^2^

1 Nuclear Medicine, Faculty of Medicine, University of Augsburg, Augsburg, Germany

2 Pathology, Faculty of Medicine, University of Augsburg, Augsburg, Germany

3 Urology, Faculty of Medicine, University of Augsburg, Augsburg, Germany

4 Hematology and Oncology, Faculty of Medicine, University of Augsburg, Augsburg, Germany

*Corresponding Author:

Dr. med. Nic Gabriel Reitsam

nic.reitsam@uka-science.de, nic.reitsam@uk-augsburg.de

**Supplementary Figures**

**Figure S1. Biomarker threshold regression model for identifying optimal cut-off of SARIFA-positive slides (percentage) for our quantitative approach.** A. Summary of biomarker threshold model with 0.3181 (31.81%) as the estimated optimal cut-off for our quantitative SARIFA approach, which we simplified to at least (≥) one third of all slides showing SARIFAs. Here, HR for quantitative SARIFA positivity according to the optimized cut-off is 1.546 (95% CI: 0.832-2.874, p = 0.168), basically identical to those of our simplified cut-off with more than one third (≥33%) of all slides. B. Plot visualizing biomarker threshold model. CI: confidence interval, HR: hazard ratio, SARIFA: Stroma-AReactive-Invasion-Front-Areas.

**Figure S2. Kaplan-Meier curves regarding overall survival of pT3a prostate cancer patients stratified by quantitative and optimized SARIFA-status.** A. SARIFA positivity (defined as ≥1/3 of all slides with SARIFAs) is not statistically associated with decreased overall survival in pT3a prostate cancer patients but showing a clear trend towards poorer outcome (p=0.16). SARIFA: Stroma-AReactive-Invasion-Front-Areas, pT: depth of invasion.

| **Supplementary Tables**  **Table S1. Clinicopathological characteristics of pT3a prostate carcinomas with regards to quantitative (≥1/3 of all slides) SARIFA-status** | | | | | | | | | |
| --- | --- | --- | --- | --- | --- | --- | --- | --- | --- |
|  |  |  |  | *SARIFA-status* | | | |  |  |
| *Variable* | | **All cases** | | **SARIFA-positive (quant.)** | | **SARIFA-negative (quant.)** | |  | |
|  |  | n=301 | 100% | n=47 | 16% | n=254 | 84% | *p-value* |  |
| **Age in years, at surgery, median (range)** | | 68 (46-87) | | 66 (52-79) | | 68 (46-87) | | 0.256 | |
| **iPSA* (mean ±standard deviation), in ng/ml** | | 14.85 ±15.19 | | 22.42 ±26.28 | | 13.40 ±11.52 | | 0.091 | |
| **pN category** | pN0 | 212 | 70% | 24 | 51% | 188 | 74% | **0.004** |  |
|  | pN1 | 87 | 29% | 23 | 49% | 64 | 25% |  |  |
|  | NA | 2 | 1% | 0 | 0% | 2 | 1% |  |  |
| **Gleason Score** | 6 | 11 | 4% | 2 | 4% | 9 | 4% | 0.101 |  |
|  | 7 | 183 | 61% | 26 | 55% | 157 | 62% |  |  |
|  | 8 | 48 | 16% | 5 | 11% | 43 | 17% |  |  |
|  | 9 | 53 | 18% | 11 | 23% | 42 | 17% |  |  |
|  | 10 | 6 | 2% | 3 | 6% | 3 | 1% |  |  |
| **Lymphatic invasion** | L0 | 216 | 72% | 29 | 62% | 187 | 74% | 0.237 |  |
|  | L1 | 26 | 9% | 6 | 13% | 20 | 8% |  |  |
|  | NA | 59 | 20% | 12 | 26% | 47 | 19% |  |  |
| **Vascular invasion** | V0 | 230 | 76% | 32 | 68% | 198 | 78% | 0.151 |  |
|  | V1 | 12 | 4% | 4 | 9% | 8 | 3% |  |  |
|  | NA | 59 | 20% | 11 | 23% | 48 | 19% |  |  |
| **Perineural invasion** | Pn0 | 8 | 3% | 1 | 2% | 7 | 3% | 0.777 |  |
|  | Pn1 | 252 | 84% | 41 | 87% | 211 | 83% |  |  |
|  | NA | 41 | 14% | 5 | 11% | 36 | 14% |  |  |
| **R status** | R0 | 172 | 57% | 19 | 40% | 153 | 60% | **0.012** |  |
|  | R1 | 129 | 43% | 28 | 60% | 101 | 40% |  |  |
| **Extraprostatic extension** | focal | 158 | 52% | 17 | 36% | 141 | 56% | **0.005** |  |
|  | non-focal | 97 | 32% | 14 | 30% | 32 | 13% |  |  |
|  | NA | 46 | 15% | 16 | 34% | 81 | 32% |  |  |
| *p*-values that are statistically significant are highlighted in **bold.** *iPSA: initial prostate specific antigen values were only available for 211 patients (34 SARIFA-positive, 177 SARIFA-negative) SARIFA: Stroma AReactive Invasion Front Areas, pT: depth of invasion, pN: lymph node status, R status: residual tumor status, quant.: quantitative approach with ≥1/3 of all slides need to show SARIFA in order to make the whole case SARIFA-positive . Extraprostatic extension according to Ball et al. ^15^. | | | | | | | | | |

|  |  |  |  | ***SARIFA-status*** | | | |  |
| --- | --- | --- | --- | --- | --- | --- | --- | --- |
| *Variable* | | All cases | | SARIFA-positive | | SARIFA-negative | |  |
|  |  | n=301 | in % | n=178 | 59% | n=123 | in % | *p-value* |
| Grade Group | 1 | 11 | 3.7 | 3 | 1.7 | 8 | 6.5 | **0.015** |
| *ISUP 2014/WHO 2016* | 2 | 88 | 29.2 | 50 | 28.1 | 38 | 30.9 |  |
|  | 3 | 95 | 31.6 | 57 | 32.0 | 38 | 30.9 |  |
|  | 4 | 48 | 15.9 | 24 | 13.5 | 24 | 19.5 |  |
|  | 5 | 59 | 19.6 | 44 | 24.7 | 15 | 12.2 |  |
| *Quantitative SARIFA assessment* | | | | SARIFA-positive (quant.) | | SARIFA-negative (quant.) | |  |
|  |  | n=301 | in % | n=47 | in % | n=254 | in % | *p-value* |
| Grade Group | 1 | 11 | 3.7 | 2 | 4.3 | 9 | 3.5 | 0.178 |
| *ISUP 2014/WHO 2016* | 2 | 88 | 29.2 | 9 | 19.1 | 79 | 31.1 |  |
|  | 3 | 95 | 31.6 | 17 | 36.2 | 78 | 30.7 |  |
|  | 4 | 48 | 15.9 | 5 | 10.6 | 43 | 16.9 |  |
|  | 5 | 59 | 19.6 | 14 | 29.8 | 45 | 17.7 |  |
|  |  |  |  |  |  |  |  |  |
| *p*-values that are statistically significant are highlighted in **bold.**  SARIFA: Stroma AReactive Invasion Front Areas quantitative (quant.) SARIFA-status: ≥1/3 of all slides with SARIFA define SARIFA-positivity | | | | | | | | |

**Table S2. Association between grade groups and SARIFA-status in pT3a grostate carcinomas**

| **Overall Survival** | ****p-*value** |
| --- | --- |
| iPSA (initial PSA value) | **0.007** |
| Age>65 | 0.379 |
| Extraprostatic extension (focal vs non-focal) | 0.105 |
| pN (pN0 vs pN1) | 0.347 |
| R status (R0 vs R1) | 0.639 |
| Lymphatic invasion (no vs yes) | 0.867 |
| Vascular invasion (no vs yes) | 0.057 |
| Perineural invasion | 0.990 |
| *Risk group | 0.466 |
| SARIFA (negative vs positive) | 0.506 |
| *p*-values that are statstically significant are higlighted in **bold.** SARIFA: Stroma AReactive Invasion Front Areas, pT: depth of invasion, pN: lymph node status, R status: residual tumor status; *Simplified risk groups are defined as: Grade Group 1 = 1; Grade Group 2 and 3 = 2; Grade Group 4 and 5 = 3. *As only the association of iPSA and survival was statistially significant in univariate Cox regression analysis, results of this multivariate model should be interpreted with caution (overfitting as potential bias!). | |

**Table S3. Multivariate Cox regression analysis regarding overall survival in pT3a prostate cancer**
